# Supplementary material for: Dopamine and fear memory formation in the human amygdala
Source: Mol Psychiatry. 2021 Dec 3;27(3):1704–11. doi: 10.1038/s41380-021-01400-x (PMC9095491; doi:10.1038/s41380-021-01400-x)
Supplement: Supplementary file 1 — Supplemental material [file 41380_2021_1400_MOESM1_ESM.docx]

Dopamine and fear memory formation in the human amygdala

Supplemental Information

**Andreas Frick**^1^, **Johannes Björkstrand**^2,3^, **Mark Lubberink**^4^, **Allison Eriksson**^5^, **Mats Fredrikson**^3,6^†, **Fredrik Åhs**^7^†

^1^The Beijer Laboratory, Department of Neuroscience, Psychiatry, Uppsala University, Uppsala, Sweden

^2^Department of Psychology, Lund University, Lund, Sweden

^3^Department of Psychology, Uppsala University, Uppsala, Sweden

^4^Department of Surgical Sciences / Nuclear Medicine & PET, Uppsala University, Uppsala, Sweden

^5^Department of Women’s and Children’s Health, Uppsala University, Sweden

^6^Department of Clinical Neuroscience, Karolinska Institutet, Stockholm, Sweden

^7^Department of Psychology and Social Work, Mid Sweden University, Östersund, Sweden

†Equal contribution

**Supplemental figures**

**Supplemental Figure 1. Bias and coefficient of variation of measures of post fear conditioning binding potential.** Bias (left) and coefficient of variation (COV; right) of binding potential (BP_ND_) values post fear conditioning based on 100 simulated time-activity curves resulting in 0-20% decrease in receptor availability, for baseline BP_ND_ of 2.6 (representing striatum), 0.3 (representing amygdala), and 0.07 (representing frontal cortex). A minor bias in post-challenge BP_ND_ of 0-2% and COV of around 10% was found for baseline BP_ND_ values of 0.3. For high baseline BP_ND_, there was a positive bias in post-challenge BP_ND_ that was proportional to dopamine release levels, and a COV of around 2.5%. For lower baseline BP_ND_, a varying bias of ±5% was seen with COV exceeding 40%.

****Supplemental Figure 2. Fitted binding potential reduction versus simulated binding potential reduction.** Binding potentials (BP_ND_) were fitted and change in BP_ND_ calculated for 100 simulated time-activity curves with 0-30% reduction in BP_ND_. Plots show fitted BP_ND_ reduction plotted against the simulated BP_ND_ reduction for the striatum, amygdala, and frontal cortex. Dashed lines represent the identity line and red solid lines the linear fit line. For baseline BP_ND_ of 2.6 (striatum), there was a negative bias in fitted BP_ND_ reduction that was proportional to dopamine release levels, in line with the positive bias in fitting post fear conditioning BP_ND_ (Supplementary Figure 2). For baseline BP_ND_ values of 0.3 (amygdala), there was a minor bias, whereas for BP_ND_ of 0.07 (frontal cortex), the scatter plot clearly illustrates the lack of reliable fit of BP_ND_ reductions at these levels.

*
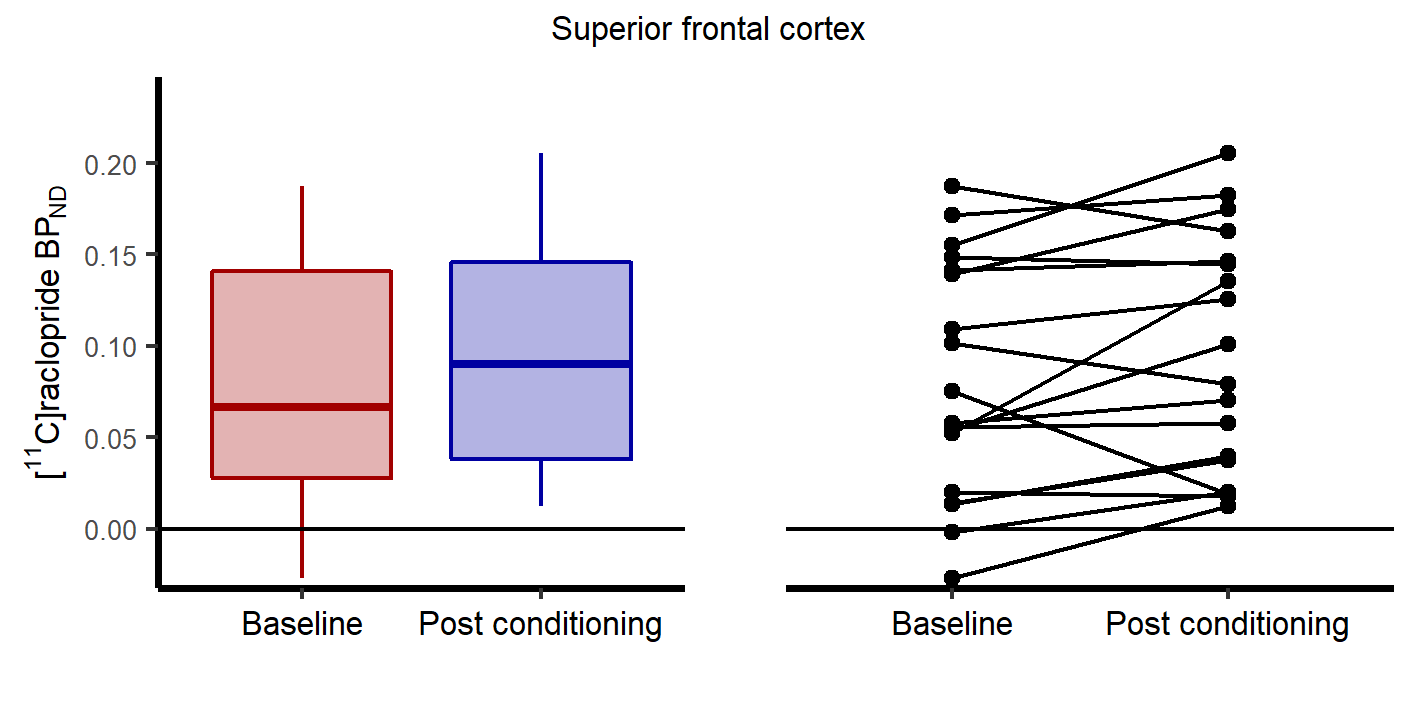
*

**Supplemental Figure 3. Binding potential of [^11^C]raclopride at baseline and after fear conditioning.** Boxplots and individual participant’s trajectory lines showing [^11^C]raclopride binding potential (BP_ND_) at baseline and after fear conditioning in anatomically defined frontal cortex (superior frontal gyrus), included here as a control region where we expected no decrease in BP_ND._ No change in BP_ND_ between baseline and post fear conditioning could be detected (mean change: -34.6%, 95% CI: -159.8% to 90.5%, t(17)=0.54, P=0.595). For the boxplots, the line indicates the median, the box the interquartile range (IQR), the whiskers the minimum of 1.5×IQR and minimum/maximum values, and circles values more extreme than 1.5×IQR. Data for individual participants is shown in the trajectory lineplots.

*
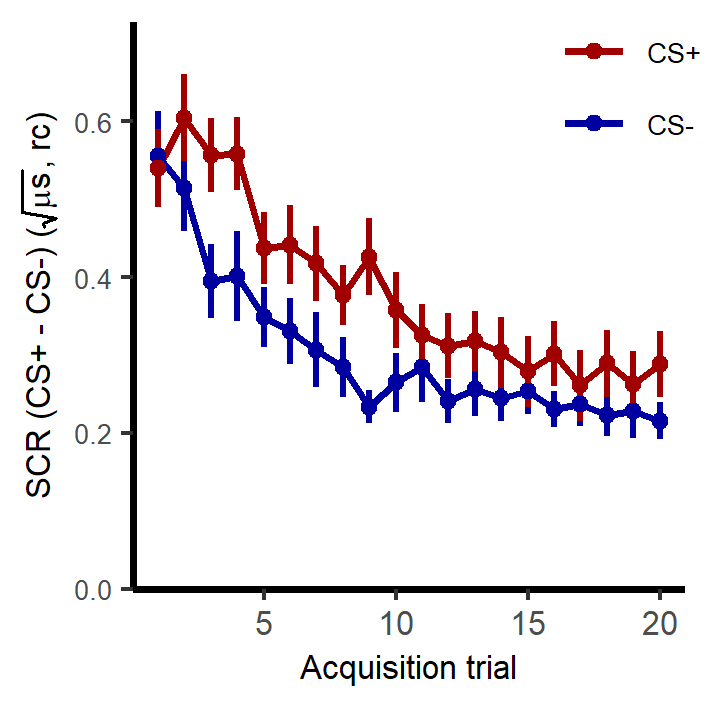
*

**Supplemental Figure 4. Trial-by-trial skin conductance responses to conditioned stimuli.** Trial-by-trial skin conductance responses (SCR) to the conditioned stimulus paired with electric shock (CS+) and the conditioned stimulus never paired with shock (CS-). A repeated measures ANOVA revealed main effects of CS (F(1, 17)=21.53, P=0.0002) and Trial (F(19, 323)=23.96, P<0.00001), and a CS × Trial interaction (F(19, 323)=2.249, P=0.0023). rc: range corrected to each individual’s maximum SCR.

*
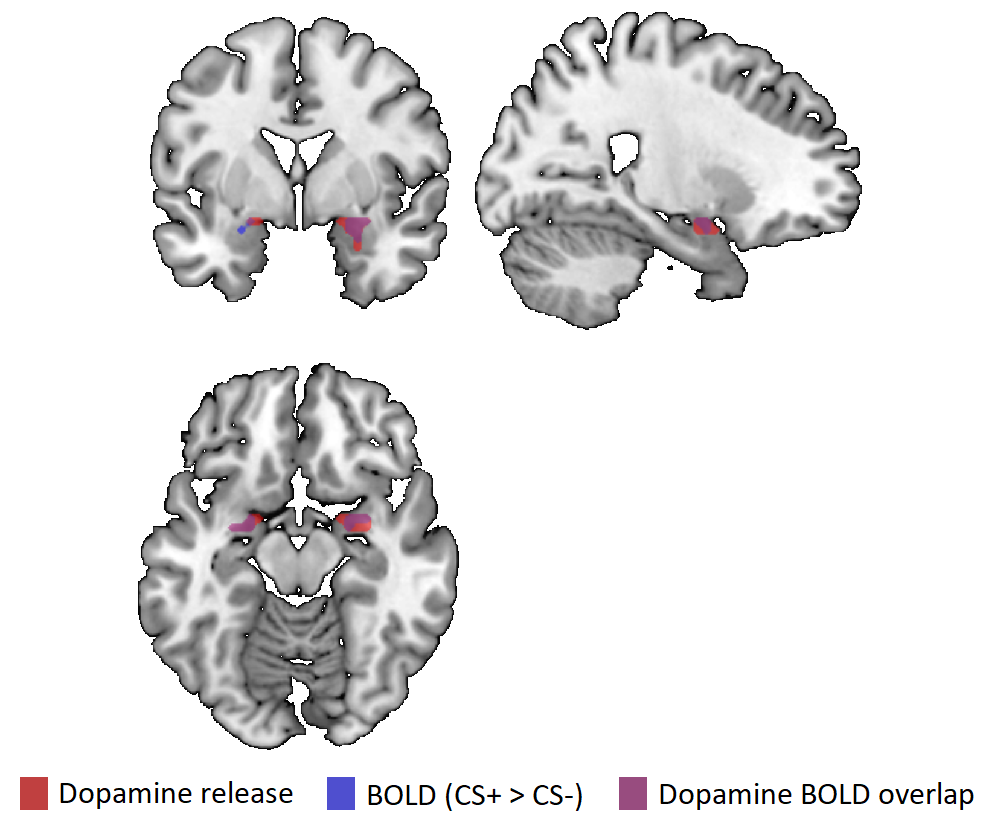
*

**Supplemental Figure 5. Dopamine release in the amygdala during fear conditioning was co-localized with the neural memory trace.** [^11^C]raclopride binding potential (BP_ND_) was decreased during fear conditioning and co-localized with the blood-oxygenation-level dependent (BOLD) response to conditioned stimuli (CS) in bilateral amygdala clusters. Red clusters show dopamine release, blue denotes clusters with greater neural activity to CS+ than CS- shown here thresholded at p<0.05 for illustrative purposes, and purple signifies overlap between dopamine release and learning-related neural activity within the amygdala. For details see Figure 4 and main text.

**Supplementary Table 1. Trial-by-trial comparisons of skin conductance responses between conditioned stimuli (CS+ and CS-).**

| **Trial** | | **F** | | **P** |
| --- | --- | --- | --- | --- |
| 1 |  | 0.155 |  | 0.698 |
| 2 |  | 2.933 |  | 0.105 |
| 3 |  | 10.835 |  | 0.004 |
| 4 |  | 13.903 |  | 0.002 |
| 5 |  | 3.647 |  | 0.073 |
| 6 |  | 8.156 |  | 0.011 |
| 7 |  | 9.610 |  | 0.007 |
| 8 |  | 6.909 |  | 0.018 |
| 9 |  | 22.157 |  | <.001 |
| 10 |  | 8.881 |  | 0.008 |
| 11 |  | 2.296 |  | 0.148 |
| 12 |  | 8.766 |  | 0.009 |
| 13 |  | 3.016 |  | 0.101 |
| 14 |  | 6.889 |  | 0.018 |
| 15 |  | 0.454 |  | 0.510 |
| 16 |  | 7.296 |  | 0.015 |
| 17 |  | 0.595 |  | 0.451 |
| 18 |  | 3.947 |  | 0.063 |
| 19 |  | 4.711 |  | 0.044 |
| 20 |  | 2.263 |  | 0.151 |
